# Multicentre cohort study evaluating clinical, oncological and functional outcomes following robotic rectal cancer surgery—the EUREKA collaborative: trial protocol

**DOI:** 10.1093/bjsopen/zrae019

**Published:** 2024-04-05

**Authors:** Christina A Fleming, Rauand Duhoky, Ritchie T J Geitenbeek, Aurore Moussion, Nabila Bouazza, Jim Khan, Eddy Cotte, Anne Dubois, Eric Rullier, Roel Hompes, Quentin Denost, Philippe Rouanet, Esther C J Consten, EUREKA collaborative, G J D van Acker, T S Aukema, H J Belgers, F H Beverdam, J G Bloemen, K Bosscha, S O Breukink, T A Burghgraef, P P L O Coene, R M P H Crolla, P van Duijvendijk, E B van Duyn, I F Faneyte, S A F Fransen, A A W van Geloven, M F Gerhards, W M U van Grevenstein, K Havenga, I H J T de Hingh, C Hoff, R Hompes, G Kats, J W A Leijtens, M F Lutke Holzik, J Melenhorst, M M Poelman, A Pronk, A H W Schiphorst, J M J Schreinemakers, C Sietses, A B Smits, I Somers, E J Spillenaar Bilgen, H B A C Stockmann, A K Talsma, P J Tanis, J Tuynman, G Verdaasdonk, P Verheijen, F A R M Warmerdam, H L van Westreenen, D D E Zimmerman

**Affiliations:** Department of Colorectal Surgery, Bordeaux Colorectal Institute, Clinique Tivoli, Bordeaux, France; Department of Colorectal Surgery, Portsmouth Hospitals University NHS Trust and the University of Portsmouth, Portsmouth, UK; Department of Surgery, University Medical Centre Groningen, Groningen, The Netherlands; Clinical Research Department, Montpellier Cancer Institute (ICM), University of Montpellier, Montpellier, France; Clinical Research Department, Montpellier Cancer Institute (ICM), University of Montpellier, Montpellier, France; Department of Colorectal Surgery, Portsmouth Hospitals University NHS Trust and the University of Portsmouth, Portsmouth, UK; Department of Digestive and Oncological Surgery, Lyon University Hospital, Lyon-Sud Hospital, Pierre-Bénite, France; Department of Colorectal Surgery, Chu Estaing, Clermont-Ferrand, France; Department of Digestive Surgery, Colorectal Unit, Haut-Lévêque Hospital, Bordeaux University Hospital, Pessac, France; Department of Surgery, Academic Medical Centre, Amsterdam, The Netherlands; Department of Colorectal Surgery, Bordeaux Colorectal Institute, Clinique Tivoli, Bordeaux, France; Surgery Department, Montpellier Cancer Institute (ICM), University of Montpellier, Montpellier, France; Department of Surgery, University Medical Centre Groningen, Groningen, The Netherlands

## Introduction

Total mesorectal excision (TME) for rectal cancer can be performed open or with minimally invasive surgery (MIS) (laparoscopic TME (L-TME), robot-assisted TME (R-TME) and transanal TME (TaTME)). Initially, when improved short-term outcomes were reported for L-TME compared with open, MIS TME was widely adopted^1–3^. However, L-TME remained challenging due to anatomical restrictions of the bony pelvis and technical limitations of laparoscopy. R-TME was developed to overcome these ergonomic limitations including a stable platform with improved precision, 3D visualization and endo-wristed instrumentation.

Initial safety and feasibility of R-TME were reported to be non-inferior but not superior compared with open and L-TME in randomized controlled trials (RCTs)^4–6^. However, trial designs have been hindered by a number of factors, including variable operative experience within robotic surgery arms and primary outcomes that may not definitively demonstrate the technical and patient-centred benefits of R-TME for rectal cancer^7^.

Furthermore, as RCTs primarily focused on oncological outcomes, there has been less emphasis on other rectal cancer outcomes including patient-reported outcome measures (PROMs). For example, does increased technical precision offer significant benefit specifically in ‘high-risk’ cases (for example, in the setting of a threatened margin (circumferential resection margin (CRM))? Can robotic surgery optimize functional outcomes^8,9^? The aim of this international multicentre idea, development, exploration, assessment and long-term follow-up (IDEAL) stage 2b collaborative work is to evaluate robotic rectal cancer surgery in the context of the above questions. The IDEAL framework lays out a systematic pathway to evaluate the safety, efficacy, and effectiveness of new surgical procedures and complex interventions^10,11^.

## Methods

### Collaborative formation

The EUREKA (Expert DUtch, FREnch and UK robotic rectal cAncer centres) collaborative was established to provide large volume ‘real-world’ data regarding robotic rectal cancer surgery. It was formed by colorectal surgeons working in high-volume robotic rectal cancer centres in France, The Netherlands and the UK. The data collection interval extends from 2013 to 2022. A full list of MIRECA and EUREKA collaborators is available in *Supplementary materials, Data S1 & S2*. The extended study protocol is available as *Data S3*.

### Study design

The study design of the EUREKA collaborative studies will be in the format of IDEAL stage 2b as a bridge from single centre to large volume multicentre observational evaluation. The full recommended criteria for performing IDEAL stage 2b studies are summarized in *Data S4*^10,11^. Studies will involve retrospective review of available data with prospective data analysis planned for future projects. It is expected that >2000 R-TME cases will be included.

### Eligibility criteria

Overall, included patients in this work will have undergone surgery after the learning curve in included centres with the following inclusion criteria also applied:

- Patients have undergone R-TME
- Biopsy-confirmed rectal cancer
- Aged 18 years or above
- Rectal tumour located within 15 cm from the anal verge.

Extended exclusion criteria are available in *Data S5*.

### Surgical interventions and perioperative oncological management

The standard oncological principles of TME were practised^12^. Choice of anastomosis and stoma use was decided based on individual patient and tumour characteristics. The following robotic surgery platforms were used in included cases: da Vinci Si (Intuitive Surgical, CA, USA) in earlier resections then subsequently da Vinci Xi (Intuitive Surgical, CA, USA). Depending on patient and tumour characteristics and institutional protocols an array of neoadjuvant and adjuvant therapies were utilized with further details in *Data S3*^13–15^.

### Definitions

A full list of study variables examined is included as *Data S6*. *Table 1* summarizes a limited list of definitions and pertinent terms used within this collaborative work.

**Table 1:** Definitions

Term	Study definition
**Oncological**	
Tumour level	Mid rectum 6–10 cm from anal verge
	Low rectum </=5 cm from anal verge
MR resection margins (including circumferential resection margin, CRM)	Negative: tumour >2 mm from resection margin
	Threatened: tumour 1–2 mm from resection margin
	Positive: tumour <1 mm from resection margin
Pathology resection margins (including CRM)	R0 tumour present >1 mm from margin
	R1 tumour present within 1 mm from margin
	R2 tumour present at resection margin
Tumour response, tumour regression grade	TRG1: complete response, no residual cancer
	TRG2: small volume residual cancer
	TRG3: fibrosis outgrowing residual cancer
	TRG4: residual cancer outgrowing fibrosis
	TRG5: absence of regression changes
EMVI	EMVI+ presence of extramural venous invasion
	EMVI– absence of extramural venous invasion
	Subclassification: small/medium/large vessel
TME quality^12^	Complete: smooth intact mesorectum, no defects >5 mm, regular CRM, no coning
	Near complete: no visible muscularis propria, irregular CRM and moderate coning
	Incomplete: defect down to muscularis propria, irregular CRM and coning
Classification of local recurrence	Anterior/central/posterior/lateral
**Clinical**	
Clavien–Dindo classification^16^	I: any deviation from normal postoperative course
	II: requiring pharmacological treatment (including blood transfusion and TPN)
	IIIa, requiring surgical endoscopic or radiological intervention; IIIb: under GA
	IV: life-threatening complication requiring ICU management
	V: death
Surgical site infection	Clinical evidence or microbiologically confirmed infection at the site of surgery
Pelvic sepsis	An umbrella term to cover anastomotic leak, pelvic abscess and peritonitis
Anastomotic leak	Loss in gastrointestinal continuity at the site of anastomosis, detected clinically, biochemically or radiologically
Anastomotic leak grading (ISREC classification)^17^	A: subclinical (managed through observation or medication)
	B: clinical (requiring radiological or transanal drainage) C: clinical (requiring re-laparotomy)
Timing of anastomotic leak	Early: < 30 days
	Late: > 30 days
Preoperative morbidity	Graded according to the ASA classification of physical health^18^
Overall survival	Defined as being alive on follow-up
Disease-free survival	Defined as being alive without recurrent disease at follow-up
Local recurrence	Defined as tumour deposit located in the pelvic cavity, with pathologically proven adenocarcinoma, or growth on consecutive imaging if histopathological confirmation was absent
Systemic recurrence	Defined as any distant metastasis, either pathologically proven or as a lesion suspect for metastasis on imaging that showed growth on consecutive imaging

MR, mesorectal; EMVI, extramural vascular invasion; TME, total mesorectal excision; TPN, total parenteral nutrition; GA, general anaesthesia; ISREC, international study group of rectal cancer.

### Patient-reported outcome measures

In this study, quality of life will be reported using both the European Organisation for Research and Treatment of Cancer (EORTC) QLQ-CR29 and QLQ-C30 scores. Bowel function will be reported using the Low Anterior Resection Score (LARS). Urinary function will be reported using the International Prostate Specific Score (IPSS). Sexual function will be reported using the International Index of Erectile Function (IIEF-5) for men and Female Sexual Function Index (FSFI) for women.

### Outcomes

The following broad project themes, in three main domains, will be investigated with a focus on complex high-risk cases and centrally placing patient-reported outcomes:

1. Cancer outcomes (for example quality of resection (CRM, R0), local recurrence, metastasis rate, disease-free survival (DFS), overall survival (OS))
2. Clinical outcomes (for example risk factors for anastomotic leak and pelvic sepsis following, definition of ‘high-risk’ patient (for example, male, high BMI, preoperative neoadjuvant therapy))
3. PROMs

### Data sharing

A principal investigator from each participating centre is responsible for quality assurance of institutional level data. A Data Sharing Agreement (DSA) was generated. In order to protect patient privacy and adhere to general data protection regulation (GDPR) (2016) guidelines, all centres pseudonymize their data and retain the pseudonymization key at their own centre, effectively making any data transfer between sites completely anonymous.

### Data management

To track all data entries the Research Data Management System (RDMS) of the University Medical Centre Groningen (UMCG) will be used to create a smaller database that will be used within this study and will fully comply with the GDPR Act, 2016. A secure digital link will be generated between the centres for data transfer. After the host (sponsoring) centre has congregated all the datasets and completed validation, verification and cleaning, the final dataset will be locked and password protected before being transferred back to the participating centres. All data queries will be submitted to and processed by the host (sponsoring) centre, and subsequently distributed to the correct participating centres. All data and documents will be archived on password-protected servers for at least 15 years by the creating party.

### Statistical analysis

At a granular level, statistical analysis will be designed based on the research question of each individualized study. For each individual study, power calculations will be performed considering difference in independent means, an s.d. of 15, a power of 0.90 and a two-sided interval. Propensity score matching may be required to overcome institutional and geographical variation. Statistical significance will be defined as a *P* value <0.05.

### Ethics and regulatory considerations

The EUREKA collaborative has received institutional review board (IRB) ethical approval from each of the participating centres for the individualized studies that have been defined and designed as part of this IDEAL stage 2b evaluation. Formal Clinical Transfer Agreements (CTA) and DSAs were completed prospectively for international data sharing.

### Role of sponsor

The sponsor (University Medical Centre Groningen) will be responsible for monitoring that the data management Standard Operating Procedure (SOP) is followed as described and will have overall responsibility for implementing systems to ensure data quality and security.

### Dissemination

The results of all studies performed by the EUREKA collaborative will be presented at relevant local, national and international scientific meetings, and will be submitted for publication in peer-reviewed journals.

## Discussion

The EUREKA collaborative aims to deliver international multicentre outcome data following robotic rectal cancer surgery, from expert centres. An IDEAL 2b study exploring outcome data from high-volume specialized centres with experienced robotic surgeons can provide valuable data to both inform practice and future research and aid in the decision-making process with patients.

**Fig 1.**
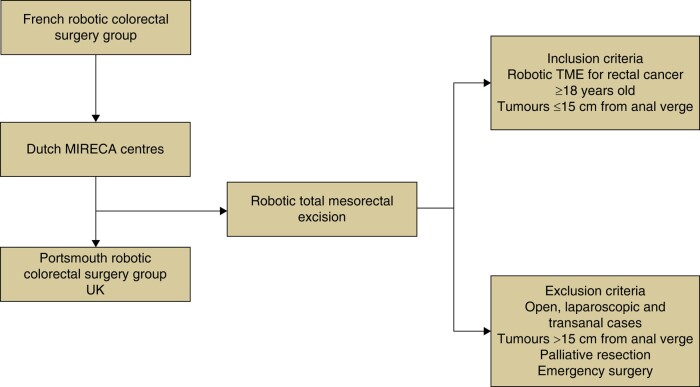
Study flow chart

TME, total mesorectal excision.

## Collaborators

G.J.D. van Acker (Haaglanden Medical Center, The Hague, The Netherlands); T.S. Aukema (Hagaziekenhuis, The Hague, The Netherlands); H.J. Belgers (Zuyderland Medical Center, Heerlen, The Netherlands); F.H. Beverdam (Fransiscus Gasthuis, Rotterdam, The Netherlands); J.G. Bloemen (Maastricht University Medical Center, Maastricht, The Netherlands); K. Bosscha (Catharina Hospital, Eindhoven, The Netherlands); S.O. Breukink (Jeroen Bosch Hospital, ‘s-Hertogenbosch, The Netherlands); T.A. Burghgraef (University Medical Center Utrecht, Utrecht, The Netherlands); P.P.L.O. Coene (Maasstad Hospital Rotterdam, Rotterdam, The Netherlands); R.M.P.H. Crolla (Amphia Hospital, Breda, The Netherlands); P. van Duijvendijk (Gelre Hospital, Apeldoorn, The Netherlands); E.B. van Duyn (Medisch Spectrum Twente, Enschede, The Netherlands); I.F. Faneyte (ZGT, Almelo, The Netherlands); S.A.F. Fransen (Maastricht University Medical Center, Maastricht, The Netherlands); A.A.W. van Geloven (Tergooi Medical Center, Hilversum, The Netherlands); M.F. Gerhards (Onze Lieve Vrouwe Gasthuis, Amsterdam, The Netherlands); W.M.U. van Grevenstein (University Medical Center Utrecht, Utrecht, The Netherlands); K. Havenga (University Medical Center Groningen, Groningen, The Netherlands); I.H.J.T. de Hingh (Catharina Hospital, Eindhoven, The Netherlands); C. Hoff (Medical Center Leeuwarden, Leeuwarden, The Netherlands); R. Hompes (Amsterdam University Medical Center, Amsterdam, The Netherlands); G. Kats (University Medical Center Groningen, Groningen, The Netherlands); J.W.A. Leijtens (Laurentius Hospital Roermond, Roermond, The Netherlands); M.F. Lutke Holzik (ZGT, Almelo, The Netherlands); J. Melenhorst (Maastricht University Medical Center, Maastricht, The Netherlands); M.M. Poelman (Fransiscus Gasthuis, Rotterdam, The Netherlands); A. Pronk (Diakonessenhuis, Utrecht, The Netherlands); A.H.W. Schiphorst (Diakonessenhuis, Utrecht, The Netherlands); J.M.J. Schreinemakers (Amphia Hospital, Breda, The Netherlands); C. Sietses (Hospital Gelderse Vallei, Ede, The Netherlands); A.B. Smits (St. Antonius Hospital, Nieuwegein, The Netherlands); I. Somers (Meander Medical Center, Amersfoort, The Netherlands); E.J. Spillenaar Bilgen (Rijnstate Hosptital, Arnhem, The Netherlands); H.B.A.C. Stockmann (Spaarne Gasthuis, Hoofddorp, The Netherlands); A.K. Talsma (Deventer Hospital, Deventer, The Netherlands); P.J. Tanis (Amsterdam University Medical Center, Amsterdam, The Netherlands); J. Tuynman (Amsterdam University Medical Center, Amsterdam, The Netherlands); G. Verdaasdonk (Jeroen Bosch Hospital, ‘s-Hertogenbosch, The Netherlands); P. Verheijen (Meander Medical Center, Amersfoort, The Netherlands); F.A.R.M. Warmerdam (Zuyderland Medical Center, Heerlen, The Netherlands); H.L. van Westreenen (Isala, Zwolle, The Netherlands); D.D.E. Zimmerman (Elisabeth TweeSteden Hospital, Tilburg, The Netherlands).

## Supplementary Material

zrae019_Supplementary_Data

## Data Availability

Anonymized data can be made available from the corresponding authors following reasonable request. In compliance with what has been agreed to in the consortium agreement and informed consent, pseudonymized data will be made accessible to other researchers through dataverse.nl (with restricted access) if they comply with Dutch legislation and comply with any restrictions that the ethics committee might impose on the reuse. To do so, researchers will have to contact the EUREKA Steering Committee.
