# Supplementary material for: Multicentre cohort study evaluating clinical, oncological and functional outcomes following robotic rectal cancer surgery—the EUREKA collaborative: trial protocol
Source: BJS Open. 2024 Apr 5;8(2):zrae019. doi: 10.1093/bjsopen/zrae019 (PMC10996923; doi:10.1093/bjsopen/zrae019)
Supplement: zrae019_Supplementary_Data [file zrae019_supplementary_data.docx]

**TITLE**

Multicentre cohort study evaluating clinical, oncological and functional outcomes following robotic rectal cancer surgery – the EUREKA collaborative: Trial Protocol

**AUTHORS**

CA Fleming^1^ *, R Duhoky^2^*, RTJ Geitenbeek^3^*, A Moussion^4^, N Bouazza^4^, J Khan^2^, E Cotte^5^, A Dubois^6^, E Rullier^7^, R Hompes^8^, Q Denost^1^ , P Rouanet^9^, ECJ Consten^3^.

On behalf of the EUREKA** collaborative

*****All authors contributed equally to writing this protocol and share first authorship

**** *E****xpert D****u****tch, F****RE****nch, and U****K*** *robotic rectal c****A****ncer centres*

**Affiliations**

^1^ Bordeaux Colorectal Institute, Clinique Tivoli, Bordeaux, France

^2^ Portsmouth Hospitals University NHS Trust and the University of Portsmouth, UK

^3^ Department of Surgery, University Medical Centre Groningen, Groningen, the Netherlands

^4^ Clinical Research Department, Montpellier Cancer Institute (ICM), Univ. Montpellier, Montpellier, France

^5^ Department of Digestive and Oncological Surgery, Lyon University Hospital, Lyon-Sud Hospital, Pierre-Bénite, France

^6^ Department of Colorectal Surgery, Chu Estaing, Clermont-Ferrand, France

^7^ Department of Digestive Surgery, Colorectal Unit, Haut-Lévêque Hospital, Bordeaux University Hospital, Pessac, France

^8^ Department of Surgery, Academic Medical Centre, Amsterdam, the Netherlands

^9^ Surgery Department, Montpellier Cancer Institute (ICM), Univ. Montpellier, Montpellier, France

**Corresponding Author**

Dr Christina Fleming

Bordeaux Colorectal Institute,

Clinique Tivoli, Bordeaux France 33000

Phone: +35361301111

Email: [christina.fleming49@gmail.com](mailto:christina.fleming49@gmail.com)

**Supplementary Materials - Index**

| **Supplementary Data 1** |  |
| --- | --- |
| Full list of MIRECA collaborators | *pag. 3* |
| **Supplementary Data 2** |  |
| Full list of EUREKA collaborators | *pag. 4* |
| **Supplementary Data 3** |  |
| Extended EURKEA study protocol | *pag. 5* |
|  |  |
| **Supplementary Data 4** |  |
| IDEAL Stage 2b framework and recommended study criteria^1,2^ | *pag. 22* |
| **Supplementary Data 5** |  |
| Full inclusion and exclusion criteria | *pag. 23* |
|  |  |
| **Supplementary Data 6** |  |
| Full list of variables recorded and studied | *pag. 24* |
| **References** | *pag. 25* |
|  |  |

**Supplementary Data 1**

**Supp Data 1:** Full list of MIRECA collaborators

G.J.D. van Acker, T.S. Aukema, H.J. Belgers, F.H. Beverdam, J.G. Bloemen, K. Bosscha, S.O. Breukink, T.A. Burghgraef, P.P.L.O. Coene, R.M.P.H. Crolla, P. van Duijvendijk, E.B. van Duyn, I.F. Faneyte, S.A.F. Fransen, A.A.W. van Geloven, M.F. Gerhards, W.M.U. van Grevenstein, K. Havenga, I.H.J.T. de Hingh, C. Hoff, R. Hompes, G. Kats, J.W.A. Leijtens, M.F. Lutke Holzik, J. Melenhorst, M.M. Poelman, A. Pronk, A.H.W. Schiphorst, J.M.J. Schreinemakers, C. Sietses, A.B. Smits, I Somers, E.J. Spillenaar Bilgen, H.B.A.C. Stockmann, A.K. Talsma, P.J. Tanis, J Tuynman, E.G.G. Verdaasdonk, P. Verheijen, F.A.R.M. Warmerdam, H.L. van Westreenen, D.D.E. Zimmerman.

**Supplementary Data 2**

**Supp. Data 2:** Full list of EUREKA Collaborators (alphabetical)

Bouazza, N (France)

Consten, E (Netherlands)

Cotte, E (France)

Denost, Quentin (France)

Dubois, A (France)

Duhoky, R (UK)

Fleming, Christina (France)

Geitenbeek, RTJ (Netherlands)

Hompes, R (Netherlands)

Khan, J (UK)

Moussion, A (France)

Rouanet, P (France)

Rullier, E (France)

**Supplementary Data 3**

**Supp Data 3:** Extended EURKEA study protocol

**TITLE**

Protocol for a multicentre cohort study evaluating clinical, oncological and functional outcomes following robotic rectal cancer surgery – the EUREKA collaborative

**AUTHORS**

CA Fleming^1^ *, R Duhoky^2^*, RTJ Geitenbeek^3^*, A Moussion^4^, N Bouazza^4^, J Khan^2^, E Cotte^5^, A Dubois^6^, E Rullier^7^, R Hompes^8^, Q Denost^1^ , P Rouanet^9^, ECJ Consten^3^.

On behalf of the EUREKA** collaborative

*****All authors contributed equally to writing this protocol and share first authorship

**** *E****xpert D****u****tch, F****RE****nch, and U****K*** *robotic rectal c****A****ncer centres*

**Affiliations**

^1^ Bordeaux Colorectal Institute, Clinique Tivoli, Bordeaux, France

^2^ Portsmouth Hospitals University NHS Trust and the University of Portsmouth, UK

^3^ Department of Surgery, University Medical Centre Groningen, Groningen, the Netherlands

^4^ Clinical Research Department, Montpellier Cancer Institute (ICM), Univ. Montpellier, Montpellier, France

^5^ Department of Digestive and Oncological Surgery, Lyon University Hospital, Lyon-Sud Hospital, Pierre-Bénite, France

^6^ Department of Colorectal Surgery, Chu Estaing, Clermont-Ferrand, France

^7^ Department of Digestive Surgery, Colorectal Unit, Haut-Lévêque Hospital, Bordeaux University Hospital, Pessac, France

^8^ Department of Surgery, Academic Medical Centre, Amsterdam, the Netherlands

^9^ Surgery Department, Montpellier Cancer Institute (ICM), Univ. Montpellier, Montpellier, France

**Corresponding Author**

Dr Christina Fleming

Bordeaux Colorectal Institute,

Clinique Tivoli, Bordeaux France 33000

Phone: +35361301111

Email: [christina.fleming49@gmail.com](mailto:christina.fleming49@gmail.com)

**Funding**

Development of this collaborative and protocol did not receive any specific funding.

**Article Category**

Study Protocol

**Previous Communication**

Nil

**Disclosure Statement**

ECJ Consten and P Rouanet are proctors for Intuitive Surgical. JSK is a proctor and trainer with Intuitive Surgical, and a trainer with Johnson & Johnson. All other authors declare: no support from any organization for the submitted work; no financial relationships with organizations that may have an interest in the submitted work in the previous 3 years; and no other relationships or activities that could appear to have influenced the submitted work.

**Data Availability Statement**

Anonymised data could be made available from corresponding author following reasonable request. In compliance with what has been agreed to in the consortium agreement and informed consent, pseudonymized data will be made accessible to other researchers through dataverse.nl (with restricted access) if they comply with Dutch legislation and comply with any restrictions that the ethics committee might impose on the reuse. In order to do so, researchers would have to contact the EUREKA Steering Committee.

**Word count:** 2640

**ABSTRACT**

**Background:** Total mesorectal excision is the standard of care for rectal cancer surgery. To date, data remains limited on robot-assisted total mesorectal excision. Therefore, this study aims to provide an IDEAL stage 2b evaluation of robotic-assisted rectal cancer surgery.

**Method:** An International collaborative group, EUREKA, was established to provide large volume ‘‘real-world’’ data regarding robotic rectal cancer surgery. Founded by experienced Colorectal Surgeons working in high volume centres using robot-assisted technique, analysis of retrospective data will be performed. Clinical, oncological, economic and patient reported outcomes will all be explored and reported, providing comprehensive data on multi-faceted outcomes following robot-assisted total mesorectal excision. Emphasis will be placed on analysing high risk complex patient cohorts that can be difficult to study due to low volume of cases in individual centres. Institutional review board ethics from each of the participating centres for the individual studies defined to date has been received and formal Clinical Transfer Agreements and Data Sharing Agreements were completed prospectively for International data sharing in compliance with the GDPR Act, 2016.

**Conclusion:** The EUREKA collaborative aims to deliver International multicentre outcome data of expert robot-assisted rectal cancer centres, necessary to provide guidance for clinical application, future research and ultimately aid in the decision making process with patients.

**Keywords:** rectal cancer; total mesorectal excision; robotic surgery; anastomotic leak; patient reported outcomes measures

**INTRODUCTION**

Primary surgical treatment of rectal carcinoma consists of resection according to the total mesorectal excision (TME) principle, which can be performed using open surgery or minimally invasive techniques as laparoscopic TME (L-TME), robot-assisted TME (R-TME), and transanal TME (TaTME)^1^. Yet no clear differences regarding intraoperative, postoperative or oncological outcomes have been described between the three techniques, when performed by experienced surgeons^2^.

As improved short-term outcomes were reported for L-TME, minimally invasive TME was widely adopted^3–5^. However, L-TME remained challenging due to anatomical restrictions of the bony pelvis and technical limitations of laparoscopy such as the fulcrum effect, rigid instruments, amplification of tremor and 2D visualization. Hence, R-TME was introduced, suggested to overcome these ergonomic limitations. Offering apparent technological advantages, a stable platform with improved precision, 3D visualisation, and endo-wristed instrumentation , R-TME was expected to improve outcomes.

Although safety and feasibility of R-TME was reported to be non-inferior compared to open and L-TME, randomised controlled trials (RCTs) found limited or no benefit of R-TME^6–8^. However, trial designs have been hindered by a number of factors, including a wide range of operative experience within robotic surgery arms and the selection of primary outcomes that may not definitively demonstrate the technical and patient-centred benefits of robotic surgery for rectal cancer^9^. As robotic surgery is still evolving worldwide, there is a wealth of experience in a number of high volume International centres, who have pioneered the advancement of robotic rectal cancer surgery. With the limitations of the current RCTs, these centres offer opportunity to comprehensively evaluate the practice of robotic rectal cancer surgery in expert hands to guide practice, future research, and to aid in the decision making process with patients.

Furthermore, as RCTs primarily focused on oncological outcomes, less attention has been paid to the effects of treatment on important outcomes such as cost-effectiveness, patient reported outcome measures (PROMs) and functional outcomes. Thus, valuable areas of rectal cancer practice have not been explored with a lack of clarity on the optimum metric of comparison. For example, does increased technical precision offered by robotic surgery platforms offer significant benefit specifically in ‘high risk’ cases (for example, in the setting of a threatened circumferential margin (CRM))? What is the benefit of enhanced recovery after surgery on cost-effectiveness^10,11^? In a digital surgery era, how can enhanced technology optimise robotic rectal cancer surgery (for example through smart stapling technology and immunofluorescence)^12–14^? How robotic surgery coupled with the enhanced technology that it supports can optimise functional outcomes^15,16^. Finally, it is postulated that robotic surgery platforms, particularly with dual console operating, can support better training and improve standardisation of surgery^17,18^.

The aim of this International multicentre IDEAL stage 2b collaborative work is to evaluate robotic rectal cancer surgery in the context of the above questions. The IDEAL framework lays out a systematic pathway to evaluate the safety, efficacy, and effectiveness of new surgical procedures and complex interventions^19,20^. An International, multicentre IDEAL stage 2b evaluation of robotic rectal cancer surgery in expert centres supports a collaborative approach to evaluate key issues that can be used to determine trial feasibility, define design features, and bridge the gap from demonstrating rationale to performing definitive comparative evaluation.

**METHODS**

**Collaborative formation**

The EUREKA (**E**xpert D**U**tch, F**RE**nch, and U**K** robotic rectal c**A**ncer centres) collaborative was established to provide large volume ‘‘real-world’’ data regarding robotic rectal cancer surgery. It was formed by Colorectal Surgeons working in high volume robotic rectal cancer centres in France, the Netherlands and the United Kingdom, who have extensive experience in rectal cancer research. The data collection period extends from 2013-2022. A full list of EUREKA collaborators is available in **Supp. Data 1**.

The aim of the EUREKA collaborative, including centres and themes, is to combine high quality data from high volume specialist robotic rectal cancer surgery centres for analysing multiple endpoints in robotic rectal cancer surgery to both add to the existing gaps in knowledge and inform future research priorities. The EUREKA collaborative aims to focus on specific complex research areas including high risk rectal cancers treated with curative intent, with a central focus on patient reported outcomes.

Dutch centres contributing to the EUREKA collaborative were comprised of the Dutch MIRECA centres, which contribute to a National, independently audited cancer registry with long-term follow-up in the Netherlands (full list of MIRECA collaborators available as **Supp. Data 2**). French data was contributed by the French collaborative group participating in ROBOT-CR study, to record outcomes and standardise care in robotic colorectal surgery practice in France. This includes four high volume rectal cancer centres: CHU Bordeaux, Institut du Cancer de Montpellier, Lyon University Hospital and CHU Estaing, Clermont-Ferrand. UK data was contributed by a high volume centre in the UK: Portsmouth Hospitals University NHS Trust (PHU) with the largest robotic rectal cancer dataset for the UK.

**Study design**

The overall study design of the EUREKA collaborative studies will be in the format of IDEAL stage 2b as a bridge from single centre to large volume multicentre observational evaluation. The full recommended criteria for performing IDEAL stage 2b studies is summarised in **Supp. Data 4.** These criteria incorporated recommendations from the original and updated IDEAL framework, including updated IDEAL recommendations for researchers and the updated IDEAL proposals for improving the surgical research environment^19,20^. Methodologically, IDEAL stage 2b criteria will be applied to and reported for each study performed. Studies will involve retrospective review of available data with prospective data analysis planned for future projects. It is expected that >2000 R-TME cases will be included.

**Eligibility criteria**

Overall, included patients in this work will have undergone surgery post the learning curve in included centres. Individual eligibility criteria will be applied for individual studies. However, patients will be included in the combined data set if the following inclusion criteria are met:

- Patients have undergone R-TME;

- Biopsy confirmed rectal cancer;

- Aged 18 years or above;

- Rectal tumour located within 15 centimetres from the anal verge.

Similarly, individual studies will apply specific exclusion criteria but patients will be excluded from the combined data set if the following exclusion criteria apply

- Patients that underwent open, L-TME and TaTME;

- Patients that underwent R-TME as part of a more complex procedure, for example a peritonectomy, or a synchronous colonic or hepatic resection;

- Patients that underwent palliative resection;

- Patients that underwent emergency resection.

**Surgical interventions and perioperative oncological management**

All centres contributing to the EUREKA collaborative perform high volume rectal cancer surgery. The standard oncological principles of TME were practiced by all participating surgeons. In brief, this involved ligation of the inferior mesorectal artery, either high or low depending on tumour location and nodal burden, and dissection of the rectum along the mesorectal plane to obtain a complete TME^21^. Choice of anastomosis, diverting stoma, or end-stoma was decided based on individual patient and tumour characteristics. Splenic flexure mobilisation was performed where deemed appropriate by the operating surgeon. All centres managed patients with an enhanced recovery after surgery (ERAS) ethos. The following robotic surgery platforms were used in included cases: da Vinci Si (Intuitive Surgical, California) in earlier resections then subsequently da Vinci Xi (Intuitive Surgical, California).

Depending on patient and tumour characteristics an array of neoadjuvant and adjuvant therapies were utilised. Commonly used neoadjuvant therapies included NACRT, SCRT, TNT (addition of induction or consolidation with NACRT or SCRT) generally using FOLFOX or FOLFIRINOX. Some geographical differences in practice between participating centres should be noted. Total neoadjuvant therapy (TNT) is quite frequently practiced in French rectal cancer centres with the use of FOLFIRINOX as the preferred systemic chemotherapy agent^22^. There is also a culture of sphincter preservation even for tumours <5cm from the anal verge with transanal low rectal dissection commonly performed to facilitate this^23^. Finally, adjuvant chemotherapy is commonly administered for ypN+ disease and is supported by National guidelines^24^. Neoadjuvant chemo- and radiotherapy is less frequently used in PHU compared to the other participating centres.

**Definitions**

A full list of study variables examined is included as **Supp. Data 6**. **Table 2** summarises a limited list of definitions and pertinent terms used within this collaborative work.

**Patient Reported Outcome Measures (PROMs)**

In this study quality of life will be reported using both the European Organisation for Research and Treatment of Cancer (EORTC) QLQ-CR29 and QLQ-C30 scores. Bowel function will be reported using the Low Anterior Resection Score (LARS). Urinary function will be reported using the International Prostate Specific Score (IPSS). Sexual function will be reported using the International Index of Erectile Function (IIEF-5) for men and Female Sexual Function Index (FSFI) for women.

**Outcomes**

To commence, the following broad project themes, in three main domains, will be investigated with a focus on complex high risk cases and centrally placing patient reported outcomes.

1. Cancer outcomes

- Quality of resection (CRM, R0)

- Predictive factors for positive circumferential resection margin rate

- Local recurrence, metastasis rate, disease free survival (DFS), overall survival (OS)

2. Clinical outcomes

- Risk factors for development of anastomotic leak and pelvic sepsis following robotic rectal cancer surgery

- Definition of High risk patient (for example, male, high BMI, pre-operative neoadjuvant therapy, T3/T4 tumours, threatened CRM, low rectal tumours <5cm, re-do surgery, etc).

3. Patient reported outcomes measures (PROMs)

- Benchmarking of PROMs following robotic rectal cancer surgery

- Analysis of the impact of pelvic sepsis on PROMs

- Analysis of the impact of HRP surgery on functional outcome (urinary, sexual, digestive) and quality of life.

**Data Sharing**

﻿A principal investigator from each participating centre is responsible for quality assurance of institutional level data. Each site identified data collector is responsible for data collection via their institutional database and submit centrally for analysis.

A Data Sharing Agreement (DSA) was generated between participating centres detailing the specifics and methods for safe data transfer. In order to protect patient privacy and adhere to GDPR (2016) guidelines, all centres pseudonymise their data and retain the pseudonymisation key at their own centre, effectively making any data transfer between sites completely anonymous. All participants will receive a research ID number, using a subject identification code list. The Subject identification code list will be maintained by the investigator, separately from the eCRF and the extracted datasets and will have secured access (PI and data manager). By using this method, the participating centres retain the ability to revisit patient records and make corrections, amendments, and additions, without transferring patient identifying information.

**Data Management**

A formal data management agreement has been agreed by all EUREKA collaborators incorporating the following principles:

**a. Data storage**

To be able to track all data entries the Research Data Management System (RDMS) of the UMCG will be used to create a smaller database that will be used within this study. This database will contain all parameters required for the current project which include, but are not limited to, patient characteristics, imaging characteristics, perioperative characteristics, histopathological characteristics and postoperative outcomes. Data storage will fully comply with the GDPR Act, 2016. As previously described, pseudo-anonymising of site-specific study data will be performed with storage of site-specific keys with the respective sites prior to data transfer essentially making transferred data anonymous.

**b. Validation and verification**

An Excel (Microsoft Office®) data template was generated to create a uniform dataset for all centres prior to data collation and transformation. This template was defined with appropriate data types and restricted values to guarantee correct data entry. After transfer, analysis will be performed to assess quality and completeness of data. Discrepancies and missing data will be reported back to participating parties to be clarified by the local investigator.

**c. Data cleaning**

Following data verification, the host centre will review the collected data and clean any apparent inconsistencies. Extensive data cleaning should not be required as it should accurately reflect electronic care records from all participating centres.

**d. Data release and transfer**

A secure digital link will be generated between the centres for data transfer. After the host (sponsoring) centre has congregated all the datasets and completed validation, verification, and cleaning, the final dataset will be locked, and password protected before being transferred back to the participating centres.

**e. Data queries**

All data queries will be submitted to and processed by the host (sponsoring) centre, and subsequently distributed to the correct participating centres. All participating centres agree to process data queries within a reasonable time frame. Data queries for individual studies will be processed by the corresponding author for each individual study.

**f. Data archiving**

Secure archive and data sharing facilities such as the Virtual research workspace are available at the UMCG. Local expertise will be provided by the data management experts of the participating institutions. All data and documents will be archived on password protected servers for at least 15 years by the creating party.

**Statistical analysis**

At a granular level statistical analysis will be designed based on the research question of each individualised study. For each individual study, power calculations will be performed considering difference in independent means, an SD of 15, a power of 0.90 and a two-sided interval. The following principles will be followed in statistical analysis: categorical variables will be analysed using Chi-squared and Fisher’s exact tests (as appropriate), continuous variables will be analysed using the Student’s t-test, Mann-Whitney-U test and Kruskal-Wallis test (as appropriate). The multivariable analysis will use logistic regression, backward selection strategy (AIC) and assessment of predictive performance. Propensity score matching may be required to overcome institutional and geographical variation. Statistical significance will be defined as a p-value <0.05.

**Ethics and regulatory considerations**

The EUREKA collaborative has received institutional review board (IRB) ethical approval from each of the participating centres for the individualised studies that have been defined and designed as part of this IDEAL stage 2b evaluation. Furthermore, formal Clinical Transfer Agreements (CTA) and Data Sharing Agreements (DSA) were completed prospectively for International data sharing.

**Role of sponsor**

The sponsor (University Medical Centre Groningen) will be responsible for monitoring that the data management Standard Operating Procedure (SOP) is followed as described and will have overall responsibility for implementing systems to ensure data quality and security.

**Dissemination**

The results of all studies performed by the EUREKA collaborative will be presented at relevant local, national, and international scientific meetings, and will be submitted for publication in peer-reviewed journals.

**DISCUSSION**

The overarching aims of the EUREKA collaborative are to deliver International expert multicentre data on robotic rectal cancer surgery clinical, oncological and patient reported outcomes. To date, randomised controlled trials (RCTs) exploring the benefits of robotic surgery for rectal cancer have been limited due to methodological challenges (for example, inclusion of learning curve cases, case heterogeneity and limited long term follow up data) and long term follow-up data is still awaited. An IDEAL 2b study exploring outcome data from high volume specialised centres with experienced robotic surgeons can provide valuable data to both inform practice and future research and aid in the decision making process with patients. Furthermore, to date data on high risk cases and centrally focusing on comparative metrics that include PROMs are lacking. The aim of this work is to specifically explore these areas and other less frequently studied and more challenging areas of evaluation, high risk rectal cancer cases and reporting of PROMs.

To commence, the EUREKA collaborative will deliver retrospective data analysis with a plan for prospective studies going forward. There may be certain data access limitations due to the retrospective nature. While outcome data is from expert centres, there will be some geographical variation in practice that may challenge outcome analysis, such as neoadjuvant treatment strategies, perioperative management and decisions regarding anastomotic techniques. These challenges will be addressed through adequate reporting and analysis of these differences between sites, and where necessary performing additional sub analyses. On the other hand, comparing QoL and functional impact from these geographic variations will prove valuable. Finally, adopting an IDEAL stage 2b approach will report on ‘real-life’ data, however, a caveat will exist that it is in experienced high-volume rectal cancer centres. As we have seen to date, however, RCTs comparing robotic surgery in rectal cancer surgery compared to other modalities have been limited, and a study analysing an accurate representation of ‘real-life’ data can significantly add to the current gap in knowledge.

In conclusion, the EUREKA collaborative aims to deliver International multicentre outcome data following robotic rectal cancer surgery, from expert centres to both inform practice and future research, and to aid in the decision making process.

**REFERENCES**

1. Heald, R., Husband, E. & Ryall, R. The mesorectum in rectal cancer surgery—the clue to pelvic recurrence? *Br. J. Surg.* **69**, 613–6. (1982).

2. Hol, J. *et al.* Comparison of laparoscopic versus robot-assisted versus transanal total mesorectal excision surgery for rectal cancer: a retrospective propensity score-matched cohort study of short-term outcomes. *Br. J. Surg.* **108**, 1380–1387 (2021).

3. van der Pas, M., Haglind, E., Cuesta, M. & et al. COlorectal cancer Laparoscopic or Open Resection II (COLOR II) Study Group Laparoscopic versus open surgery for rectal cancer (COLOR II): short-term outcomes of a randomised, phase 3 trial. *Lancet Oncol.* **14**, 210–218 (2013).

4. Park, J. *et al.* Open versus laparoscopic surgery for mid or low rectal cancer after neoadjuvant chemoradiotherapy (COREAN trial): 10-year follow-up of an open-label, non-inferiority, randomised controlled trial. *Lancet Gastroenterol. ad Hepatol.* **6**, 569–577 (2021).

5. Stevenson, A. R. L. *et al.* Effect of laparoscopic-assisted resection vs open resection on pathological outcomes in rectal cancer: The ALaCaRT randomized clinical trial. *JAMA - J. Am. Med. Assoc.* **314**, 1356–1363 (2015).

6. Jayne, D. *et al.* Effect of robotic-assisted vs conventional laparoscopic surgery on risk of conversion to open laparotomy among patients undergoing resection for rectal cancer the rolarr randomized clinical trial. *JAMA - J. Am. Med. Assoc.* **318**, 1569–1580 (2017).

7. Kim, M. J. *et al.* Robot-assisted Versus Laparoscopic Surgery for Rectal Cancer: A Phase II Open Label Prospective Randomized Controlled Trial. *Ann. Surg.* **267**, 243–251 (2018).

8. Feng, Q. *et al.* Robotic versus laparoscopic surgery for middle and low rectal cancer (REAL): short-term outcomes of a multicentre randomised controlled trial. *Lancet Gastroenterol. ad Hepatol.* **7**, 991–1004 (2022).

9. Corrigan, N. *et al.* Exploring and adjusting for potential learning effects in ROLARR: A randomised controlled trial comparing robotic-assisted vs. standard laparoscopic surgery for rectal cancer resection. *Trials* **19**, 1–11 (2018).

10. Harji, D. *et al.* A novel bowel rehabilitation programme after total mesorectal excision for rectal cancer: the BOREAL pilot study. *Color. Dis.* **23**, 2619–2626 (2021).

11. Rouanet, P. *et al.* Combined robotic approach and enhanced recovery after surgery pathway for optimization of costs in patients undergoing proctectomy. *BJS open* **4**, 516–523 (2020).

12. Tejedor, P., Sagias, F., Flashman, K., Kandala, N. L. & Khan, J. The use of robotic or laparoscopic stapler in rectal cancer surgery: a systematic review and meta-analysis. *J. Robot. Surg.* **14**, 829–833 (2020).

13. Tejedor, P. *et al.* Advantages of using a robotic stapler in rectal cancer surgery. *J. Robot. Surg.* **14**, 365–370 (2020).

14. Tejedor, P., Sagias, F. & Khan, J. The Use of Enhanced Technologies in Robotic Surgery and Its Impact on Outcomes in Rectal Cancer: A Systematic Review. *Surg. Innov.* **27**, 384–391 (2020).

15. Fleming, C. A. *et al.* Urogenital function following robotic and laparoscopic rectal cancer surgery: meta-analysis. *Br. J. Surg.* **108**, 128–137 (2021).

16. Kim, H. J. *et al.* The impact of robotic surgery on quality of life, urinary and sexual function following total mesorectal excision for rectal cancer: a propensity score-matched analysis with laparoscopic surgery. *Color. Dis.* **20**, O103–O113 (2018).

17. Tou, S. *et al.* European expert consensus on a structured approach to training robotic-assisted low anterior resection using performance metrics. *Color. Dis.* 2232–2242 (2020). doi:10.1111/codi.15269

18. Eardley, N. *et al.* European Society of Coloproctology Colorectal Robotic Surgery Training for the Trainers Course - the first pilot experience. *Color. Dis.* **22**, 1741–1748 (2020).

19. Agha, R. A., Hirst, A., Khachane, A. & McCulloch, P. A protocol for the development of reporting guidelines for IDEAL stage studies. *Int. J. Surg. Protoc.* **9**, 11–14 (2018).

20. Hirst, A. *et al.* No Surgical Innovation Without Evaluation: Evolution and Further Development of the IDEAL Framework and Recommendations. *Ann. Surg.* **269**, 211–220 (2019).

21. Nagtegaal, I., van de Velde, C., van der Worp, E. & Al., E. Macroscopic evaluation of rectal cancer resection specimen: clinical significance of the pathologist in quality control. *J. Clin. Oncol.* **20**, 1729–1734 (2002).

22. Conroy, T. *et al.* Neoadjuvant chemotherapy with FOLFIRINOX and preoperative chemoradiotherapy for patients with locally advanced rectal cancer (UNICANCER-PRODIGE 23): a multicentre, randomised, open-label, phase 3 trial. *Lancet Oncol.* **22**, 702–715 (2021).

23. Denost, Q. *et al.* Intersphincteric resection for low rectal cancer: the risk is functional rather than oncological. A 25-year experience from Bordeaux. *Color. Dis.* **22**, 1603–1613 (2020).

24. Denost, Q. *et al.* An international multicenter prospective study evaluating the long-term oncological impact of adjuvant chemotherapy in ypN+ rectal cancer. *Ann. Surg.* **Oct 28.**, Epub ahead of print. (2022).

25. Dindo, D., Demartines, N. & Clavien, P. Classification of surgical complications: a new proposal with evaluation in a cohort of 6336 patients and results of a survey. *Ann. Surg.* **240**, 205–213 (2004).

26. Kulu, Y. *et al.* International Study Group of Rectal Cancer. Validation of the International Study Group of Rectal Cancer definition and severity grading of anastomotic leakage. *Surgery* **153**, 753–761 (2013).

27. Daabiss, M. American society of anaesthesiologists physical status classification. *Indian J. Anaesth.* **55**, 111–115 (2011).

**ETHICS STATEMENT**

The EUREKA collaborative has received institutional review board (IRB) ethics from each of the participating centres for the individualised studies that have been defined and designed as part of this IDEAL stage 2b evaluation. Furthermore, formal Clinical Transfer Agreements (CTA) and Data Sharing Agreements (DSA) were completed prospectively for international data sharing.

**AUTHOR CONTRIBUTIONS**

Fleming/Duhoky/Geitenbeek: All authors contributed equally and share first authorship.

Substantial contributions to the conception and design of the work: Fleming, Duhoky, Geitenbeek, Moussion, Bouazza, Khan, Cotte, Dubois, Rullier, Hompes, Denost, Rouanet, Consten

Drafting the article: Fleming, Duhoky, Geitenbeek.

Revising the article critically for important intellectual content: Khan, Denost, Rouanet, Consten.

Final approval of the version to be published: Fleming, Duhoky, Geitenbeek, Moussion, Bouazza, Khan, Cotte, Dubois, Rullier, Hompes, Denost, Rouanet, Consten.

All authors agree to be accountable for all aspects of the work in ensuring that questions related to the accuracy or integrity of any part of the work are appropriately investigated and resolved. The EUREKA collaborative consists of all local investigators responsible for patient recruitment in their respective centres. They all provided insight and feedback during the conception and design of the work.

**Amendments**In the event of protocol amendments, the date of amendment and rationale for deviation will be provided. Issue date: **-**-2022. Protocol amendment number: N/A.

**FIGURES**

**Figure 1:** Study flowchart

**
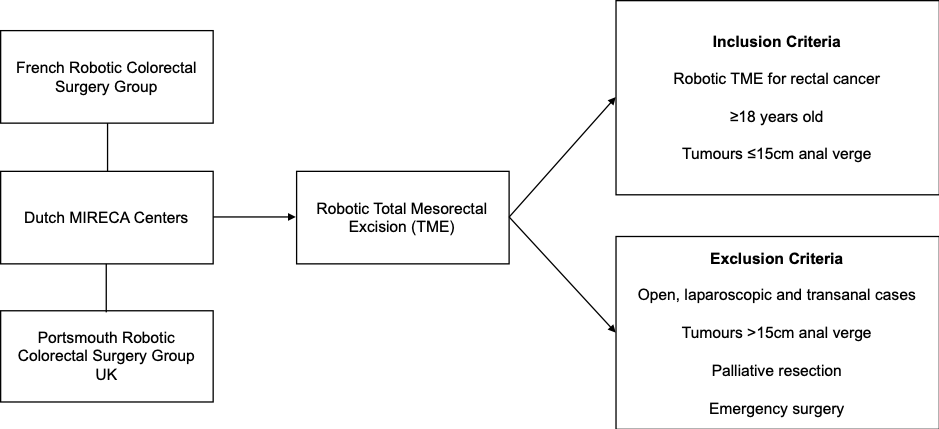
**

**TABLES**

**Table 1:** Eligibility criteria for inclusion in the combined Collaborative data (individual study eligibility criteria will also be applied).

TME: total mesorectal excision. R: robotic. L: laparoscopic.

| **Inclusion Criteria** |
| --- |
| 1. Patients have undergone a R-TME procedure 2. Biopsy confirmed rectal cancer 3. Aged 18 years or above 4. Rectal tumour located within 15 centimetres from the anal verge |
| **Exclusion Criteria** |
| 1. Open, L-TME and TaTME 2. R-TME performed as part of a more complex procedure, for example a peritonectomy, or a synchronous colonic or hepatic resection 3. Palliative resection 4. Emergency resection |

**Table 2:** Definitions

| **TERM** | **STUDY DEFINITION** |
| --- | --- |
| **ONCOLOGICAL** | **Tumour Level**  Mid rectum 6-10cm from anal verge  low rectum </=5cm from anal verge  **MR Resection Margins (including circumferential resection margin, CRM)**  Negative: tumour >2mm from resection margin  Threatened: tumour 1-2mm from resection margin  Positive: tumour <1mm from resection margin  **Pathology Resection Margins (including CRM)**  R0 tumour present >1mm from margin  R1 tumour present within 1mm from margin  R2 tumour present at resection margin  **Tumour Response, Tumour Regression Grade (TRG)**  TRG1: complete response, no residual cancer  TRG2: small volume residual cancer  TRG3: fibrosis outgrowing residua cancer  TRG4: residual cancer outgrowing fibrosis  TRG5: absence of regression changes  **EMVI**  EMVI+ presence of extramural venous invasion  EMVI- absence of extramural venous invasion  Sub-classification: small / medium / large vessel  **TME Quality**^21^  Complete: smooth intact mesorectum, no defects >5mm, regular CRM, no coning  Near complete: no visible muscularis propria, irregular CRM and moderate coning  Incomplete: defect down to muscularis propria, irregular CRM and coning  **Classification of Local Recurrence**  Anterior / Central / Posterior / Lateral |
| **CLINICAL** | **Clavien-Dindo Classification**^25^  I, any deviation from normal postoperative course  II, requiring pharmacological treatment (including blood transfusion and TPN)  IIIa, requiring surgical endoscopic or radiological intervention; IIIb, under GA  IV, life-threatening complication requiring ICU management  V, death  **Surgical Site Infection**  Clinical evidence or microbiologically confirmed infection at the site of surgery  **Pelvic Sepsis**  An umbrella term to cover anastomotic leak, pelvic abscess and peritonitis  **Anastomotic Leak**  Loss in gastrointestinal continuity at the site of anastomosis, detected clinically, biochemically or radiologically  **Anastomotic Leak Grading (ISREC Classification)**^26^  A: subclinical (managed through observation or medication)  B: clinical (requiring radiological or transanal drainage)  C: clinical (requiring re-laparotomy)  **Timing of Anastomotic Leak**  Early: <30 days  Late: >30 days  **Preoperative morbidity**  Graded according to the American Society of Anaesthesiologists’ (ASA) classification of Physical Health^27^.  **Overall survival**  Defined as being alive follow-up.  **Disease-free survival**  Defined as being alive without recurrent disease at follow-up.  **Local recurrence**  Defined as tumour deposit located in the pelvic cavity, with pathological proven adenocarcinoma, or growth on consecutive imaging if histopathological confirmation was absent.  **Systematic recurrence**  Defined as any distant metastasis, either pathologically proven or as a lesion suspect for metastasis on imaging that showed growth on consecutive imaging. |

**Supplementary Data 4**

**Supp Data 4:** IDEAL Stage 2b framework and recommended study criteria^1,2^

| **IDEAL Stage 2b Framework and Criteria** |
| --- |
| **IDEAL Framework** |
| **Purpose:** Achieving consensus between surgeons and centres  **Number and Types of Patients:** Many; broadening indication to include all potential beneficiaries  **Number and Types of Surgeons:** Many; innovators, early adopters, early majority  **Output:** Effect estimate based on large sample; Analysis of learning curves; estimate of influence of prespecified technical variants and patient subgroups on outcome.  **Intervention:** Stable; acceptable variants defined    **Method:** Prospective multicentre exploration cohort study (disease or treatment based); pilot/feasibility multicentre RCTs.    **Outcomes:** Safety; clinical outcomes (specific/ graded); short-term outcomes; patient cantered/ reported outcomes; feasibility outcomes    **Stage Endpoints:** fall in to two main groups; Demonstrate that technique can be more widely adopted; and Demonstrate that progression to RCT is desirable and feasible |
| **Recommendations for Researchers** |
| 1. Make protocol for study available    2. Use standard well-defined measures for reporting outcome and patient characteristics    3. Participate in collaborative multi-centre cooperative data collection, incorporating feasibility issues such as:                                    a. estimating effect size                                    b. defining intervention quality standards                                    c. evaluating learning curves                                    d. exploring subgroup differences                                    e. eliciting key stakeholder values and preferences                                  f. analysis of adverse events:    4. Pre-planned consensus meeting prior to progressing to an RCT to identify feasibility and ability to recruit, intervention and comparator definitions, appropriate patient selection criteria, primary endpoint. |
| **Recommendations for Improving the Surgical Research Environment** |
| **Funders:** Support Stage 2b Exploratory cohort studies as preliminary ‘‘pilot/ feasibility’’ phases for RCT proposals.    **Journals:** Support publication of IDEAL Exploration studies and protocols    **Ethical aspects:** formal human research ethics approval required Ensure that potential harms from the learning curve are minimized by training and mentoring prior to progressing to Stage 3 |

**Supplementary Data 5**

**Supp. Data 5:** Eligibility criteria for inclusion in the combined Collaborative data (individual study eligibility criteria will also be applied).

TME: total mesorectal excision. R: robotic. L: laparoscopic.

| **Inclusion Criteria** |
| --- |
| 1. Patients have undergone a R-TME procedure 2. Biopsy confirmed rectal cancer 3. Aged 18 years or above 4. Rectal tumour located within 15 centimetres from the anal verge |
| **Exclusion Criteria** |
| 1. Open, L-TME and TaTME 2. R-TME performed as part of a more complex procedure, for example a peritonectomy, or a synchronous colonic or hepatic resection 3. Palliative resection 4. Emergency resection |

**Supplementary Data 6**

**Supp. Data 6:** Full list of variables recorded and studied

| Sex | Surgery Type | Time to discharge (days) | Histology |
| --- | --- | --- | --- |
| Age | Intervention | Complications | Specimen Quality / grade of TME |
| ASA score | Elective | Complication within 30 days | TNM-T |
| BMI | Operating time | Time to pelvic sepsis | C_pT4 |
| Diabetic | Docking time (mins) | Complication Leak | pTMN |
| Cardiovascular | Conversion? | ISREC classification leak | pTMN |
| Previous Surgery | Conversion type | Early or late leak | Number of nodes harvested |
| Status Metastatic | Conversion reason | Pelvic Sepsis up to 30 days postop | Nodes Positive |
| Stoma prior to surgery | Anastomosis | Peritonitis | Resection |
| Death | Stoma | Pelvic sepsis treatment | Circumferential margin involved |
| Cause Of Death | Stoma reversal/closure | Other postop complications | Distance from CRM to tumour margin in mm on pathology |
| Death Cancer Related | Was a stapler used in procedure? | Clavien-Dindo | Closest distal resection margin (mm) |
| Time from diagnosis to death (days) | Stapler Type | Date of re-intervention | Perforation |
| Overall survival (days) | What was the linear stapler used for in the procedure? | Reintervention within 31 days postop? | Local Recurrence |
| Disease free survival (days) | Stapler length used? | Type of re-intervention | Time to local recurrence (days) |
| Pre-operative MRI | Type of reload | Date of readmission | Local Recur Location |
| T stage (Before chemoradiotherapy) | Number of reload | Readmission within 31 days postop | Distant metastases? |
| Subclassification T4 | Number of Robotic Stapler fires | Date of follow-up | Time to metastases (days) |
| N stage (before CT/RT) | Number of Robotic Stapler misfires | Adjuvant Chemotherapy | Systemic Recur Location |
| Preop Mets | Drain | Lost to follow-up | PROMs |
| Biopsy Histology | Fluorescence Imaging System used | Stoma in follow-up | QLQ- CR- 29 |
| MRI tumour height | Intra-operative Complication(s) | Stoma complication | QLQ-C30 |
| MRI EMVI | Intra-operative bleeding | Stoma revision | FSFI |
| Location in the lumen | IntraOpPerforation | Stoma Reversal | IPSS |
| MRF(mm) Before chemoradiotherapy | Other Intra-operative complications |  | IIEF5 |
| Tumour size(cm) Before CRT |  |  | LARS |
| Tumour size (cm) After CRT |  |  |  |
| NeoAdjuvant therapy |  |  |  |
| Tumour response |  |  |  |
| yciT(after CRT) |  |  |  |
| yciT4 substage |  |  |  |
| C_ycN |  |  |  |
| MRF post nCRT (cm) |  |  |  |

**References**

1. Agha, R. A., Hirst, A., Khachane, A. & McCulloch, P. A protocol for the development of reporting guidelines for IDEAL stage studies. *Int. J. Surg. Protoc.* **9**, 11–14 (2018).

2. Hirst, A. *et al.* No Surgical Innovation Without Evaluation: Evolution and Further Development of the IDEAL Framework and Recommendations. *Ann. Surg.* **269**, 211–220 (2019).
